# Self-medication with non-prescribed pharmaceutical agents in an area of low malaria transmission in northern Tanzania: a community-based survey

**DOI:** 10.1093/trstmh/try138

**Published:** 2018-12-31

**Authors:** Julian T Hertz, Deng B Madut, Revogatus A Tesha, Gwamaka William, Ryan A Simmons, Sophie W Galson, Venance P Maro, John A Crump, Matthew P Rubach

**Affiliations:** 1 Department of Surgery, Division of Emergency Medicine, Duke University Medical Center, 2301 Erwin Rd, Durham, NC, USA; 2 Department of Medicine, Division of Infectious Disease, Duke University Medical Center, 2301 Erwin Rd, Durham, NC, USA; 3 Department of Statistical Science, Duke University, Durham, NC, USA; 4 Kilimanjaro Christian Medical Centre, Moshi, Tanzania; 5 Duke Global Health Institute, Duke University, 310 Trent Dr, Durham, NC, USA; 6 Otago Global Health Institute, University of Otago, Dunedin, New Zealand

**Keywords:** antihypertensive agents, anti-infective agents, self-medication, sub-Saharan Africa, Tanzania

## Abstract

**Background:**

Self-treatment with antimicrobials is common in sub-Saharan Africa. Little is known about the prevalence of this practice where malaria transmission intensity is low, and little is known about the prevalence of self-treatment with other medications such as antihypertensives and antihyperglycemics.

**Methods:**

A two-stage randomized population-based cluster survey with selection proportional to population size was performed in northern Tanzania. Self-identified healthcare decision-makers from randomly selected households were asked to report instances of self-medication without a prescription in the preceding year. Associations between self-treatment and sociodemographic characteristics were assessed with Pearson’s chi-squared and the Student’s t-test.

**Results:**

A total of 718 participants completed the survey, and 344 (47.9%) reported any household member obtaining medication without a prescription. Of these, 85 (11.8%) obtained an antimicrobial and four (0.6%) obtained an antihypertensive or antihyperglycemic. Of respondents reporting self-treatment, 306 (89.0%) selected the medication themselves. Self-treatment with antimicrobials was associated with post-primary education (OR 1.95, 95% CI 1.22–3.16, *p*=0.005), younger age (43.1 vs 48.7 years, *p*=0.007) and higher socioeconomic status score (0.42 vs 0.34, *p*=0.023).

**Conclusions:**

Self-treatment with antimicrobials in an area of low malaria transmission intensity was uncommon and self-treatment with antihypertensives and antihyperglycemics was rare.

## Introduction

Self-treatment with antimicrobials obtained without a prescription is common in sub-Saharan Africa. In recent community-based surveys conducted in Cameroon,^1^ Ethiopia,^2^ Ghana,^3^ Ivory Coast,^4^ Sudan^5^ and Uganda,^6^ between 39–80% of respondents admitted to recent self-treatment with an antimicrobial. The use of antimicrobials without a prescription has drawn the interest of public health officials primarily due to concerns that such inappropriate use may be contributing to growing prevalence of antibacterial and antimalarial resistance.^7,8^ These concerns have resulted in calls for better regulation of pharmaceutical dispensing across the continent. Less is known about the prevalence of antimicrobial self-treatment in Tanzania than in other countries in sub-Saharan Africa. However, preliminary studies have demonstrated high levels of willingness among local pharmacists to dispense antimicrobials without a prescription, despite Tanzanian laws which require prescriptions for all antibacterials and antimalarials.^9,10^

Beyond characterizing the specific prevalence of self-treatment in Tanzania, additional important questions about the use of non-prescribed pharmaceutical agents in sub-Saharan Africa remain unanswered. First, little research has been done to identify the recommenders of specific pharmaceutical agents. Although the use of antimalarials without a prescription is common in sub-Saharan Africa,^1,11^ less is known about who specifically decides that antimalarial treatment is indicated without a clinician’s prescription. Knowledge of the recommenders’ identities will help tailor effective interventions to combat inappropriate use of prescription medications in sub-Saharan Africa. For example, different interventions would be warranted if the primary selectors of specific medications were patients vs pharmacists.

A second important gap in understanding self-treatment in the region is to understand how self-treatment patterns differ in areas of low-malaria transmission. The majority of studies regarding antimicrobial use have been conducted in areas where prevalence of malaria is high.^1,7,11^ As malaria prevalence declines across sub-Saharan Africa,^12^ increasing numbers of people will be residing in areas of low-malaria transmission; but it is unknown how antimicrobial self-treatment behaviors differ in such areas.

There has also been sparse study of self-treatment with non-antimicrobial medications such as antihypertensives and antihyperglycemics. As hypertension and diabetes become increasingly prevalent across sub-Saharan Africa,^13–15^ there is likely to be increasing demand for medications for these chronic conditions. Self-treatment for hypertension and diabetes would be concerning given the need for careful consideration of side effects, comorbidities and dose adjustments.

The objectives of this study were the following: to estimate the prevalence of self-treatment with antimicrobials, antihypertensives and antihyperglycemics in an area of low malaria transmission intensity, to identify risk factors for self-treatment, and to identify recommenders of specific medications. To do so, we conducted a cross-sectional community survey of adults in the Kilimanjaro Region.

## Materials and methods

### Study setting

The study was conducted from February–May 2018 in three districts of the Kilimanjaro Region of northern Tanzania: the urban district of Moshi Urban (population 184 289) and two surrounding rural districts, Moshi Rural and Hai (populations of 466 740 and 210 531, respectively).^16^ The local malaria prevalence is low; a recent survey found the prevalence of malaria among children under 5 years of age to be between 0.0–0.5%.^17^ The dominant local tribe is the Chagga tribe.

### Participant selection

A two-stage randomized population-based cluster survey was performed with selection proportional to population size, following WHO recommendations for vaccination coverage cluster surveys.^18^ Sixty sub-districts in the study districts were chosen randomly in a population-weighted fashion, with urban and rural settings selected proportionally to their population size. The 60 sub-districts selected for study inclusion are depicted in Supplemental Figure 1. A starting point in each selected sub-district was chosen randomly using Quantum Geographic Information System (QGIS, v2.18.7). A one square km polygon was drawn around this starting point and 12 random coordinates were selected within this polygon. These coordinates were visited on foot by study staff using the Garmin eTrex geographical positioning system (Garmin, Olathe, KS, USA) and the nearest household was selected for survey inclusion. Surveys were conducted with any individual who identified themselves as a healthcare decision-maker for the household, with 12 households surveyed per sub-district. Informed consent was obtained from all participants prior to initiation of the surveys. Inclusion criteria for survey participation were residence in the selected household, self-identification as a healthcare decision-maker for the household, and ability to provide informed consent. If no eligible participant was available, the next nearest household was approached for inclusion.

### Study procedures

Surveyors conducted the surveys in Swahili on tablets (Samsung Galaxy A, Samsung, Seoul, Korea) using Open Data Kit software (ODK v1.12.2, Seattle, WA, USA). Participants were asked whether or not they or anyone else in the house had self-treated with any medication without a prescription in the past 12 months. They were further asked to estimate the total number of times they had obtained medications without a prescription and to identify what kind of medication was obtained in these instances, if known. Respondents selected the type of medication from a standardized picklist, and surveyors were trained to assist with this question when respondents had trouble identifying the appropriate medication type. Participants were also asked to report where they would seek care if a member of their household were to have a febrile illness. Demographic information including age and gender of the respondent as well as the education level of the head of household was also collected.

### Data analysis

Summary statistics for categorical variables were reported as proportions, and for continuous variables were reported as medians and ranges, or means and SDs. Pearson’s chi-square was used to analyze the association between self-treatment with antimicrobials and certain categorical respondent characteristics; the t-test was used to compare means between groups. ORs and corresponding CIs were calculated from contingency tables. The t-test was performed in STATA (v15.1); all other analyses were performed in R (v3.3.2). All analyses were performed with a 0.05 cut-off level for statistical significance. Antimicrobial medication was defined as either antibacterial, antimalarial or antihelmintic. Urban residence was defined as residence in Moshi Urban District. ‘Preferring pharmacy for febrile syndrome’ was defined as identifying a pharmacy as the first choice facility for either a male or a female with a fever. A socioeconomic status (SES) score was derived via principal component analysis^19^ using nine binary variables: post-primary education, presence of electricity in the home, health insurance coverage, home floor material, ownership of a bank account, ownership of a car, ownership of a television, ownership of a refrigerator and presence of a flush toilet in the home. Participant age, gender, religion, tribal affiliation, health insurance ownership and education level were defined by participant self-report. The minimum sample size was computed assuming a proportion (defined as the study area’s population that sought healthcare for fever at a specific healthcare facility) of 0.1, a precision of 10%, a design effect of 1.5 and an inflation factor of 1.2. The sample size calculation resulted in a minimum of 60 sub-districts to be visited with 11 households surveyed from each village. Sub-district population data were taken from the 2012 Tanzania Population and Housing Census.^16^

### Research ethics

This study received ethics approval from the Duke Health Institutional Review Board (protocol Pro00016134), the Kilimanjaro Christian Medical Centre Research Ethics Committee (protocol number 295) and the Tanzania National Institutes for Medical Research Ethics Coordinating Committee (protocol NIMR/HQ/R.8a/Vol. IX/1000).

## Results

Of 718 individuals participating in the survey, 485 (67.5%) were females. The median (range) age of participants was 48 (17–99) years and 230 (32.0%) households had health insurance. Sociodemographic characteristics of survey respondents are presented in Table 1.

**Table 1.** Sociodemographic features of household survey respondents, Moshi Urban, Moshi Rural and Hai Districts, 2018 (*N*=718)

	n	(%)
Female	485	(67.5)
Urban residence	155	(21.6)
Education		
None	40	(5.6)
Primary	497	(69.2)
Secondary	132	(18.4)
Post-Secondary	49	(6.8)
Have health insurance	230	(32.0)
Religion		
Christian	584	(81.3)
Muslim	115	(16.0)
Other	19	(2.6)
Chagga tribe	535	(74.5)
	Median	(Range)
Age, years	48	(17, 99)
Household size, number of persons	4	(1, 13)
SES score	0.29	(0, 1.01)

SES: socioeconomic status

Of participants, 344 (47.9%) reported that they or someone in their household had obtained a medication without a prescription in the past 12 months. Of those obtaining pharmaceutical agents without a prescription, the median (range) number of times they reported doing so over the preceding year was 4 (1, 98). Table 2 lists the types of medications respondents reported obtaining without prescriptions. Analgesics were the most commonly identified type, obtained by 315 (43.9%) of respondents. Eighty-five (11.8%) participants reported obtaining any antimicrobial including antibacterials, antimalarials or antihelmintics without a prescription. Of the antimicrobials, antihelmintics were the most commonly reported, obtained by 54 (7.5%). Four (0.6%) respondents reported obtaining either antihypertensive or antihyperglycemic medications without a prescription in the preceding year.

**Table 2.** Types of medications obtained without a prescription by residents of Moshi Urban, Moshi Rural and Hai Districts over the preceding 12 months, 2018 (*N*=718)

Medication type	n	(%)
None	374	(52.1)
Analgesic	315	(43.9)
Cold and cough	98	(13.6)
Antihelmintic	54	(7.5)
Herbal	40	(5.6)
Antimalarial	37	(5.2)
Antibacterial	13	(1.8)
Antihypertensive	2	(0.3)
Antihyperglycemic	2	(0.3)
Other	3	(0.4)

Table 3 presents the recommenders or selectors of specific medications when they were obtained without a prescription. Of respondents who disclosed obtaining medications without prescriptions, 306 (89.0%) reported that they selected the medication themselves.

**Table 3.** Recommenders of specific medications when medications were obtained without a prescription in northern Tanzania, 2018 (*N*=344)

Recommender	*n*	(%)
Self	306	(89.0)
Friend	16	(4.7)
Doctor	13	(3.8)
Pharmacist	9	(2.6)
Family member	3	(0.9)

Table 4 compares the sociodemographic features of those who reported obtaining an antimicrobial medication without a prescription and those who did not. Those who obtained antimicrobials without a prescription were more likely to have post-primary education (OR 1.96, *p*=0.005), were more likely to be younger (*p*=0.007) and were more likely to have higher SES (*p*=0.023) than those who did not. There was no association between obtaining antimicrobials without a prescription and female gender, urban residence, health insurance ownership, religion or tribal affiliation. Respondents who reported self-treatment with antimicrobials were significantly more likely than those who did not to prefer seeking healthcare directly at pharmacies for febrile illnesses (OR 1.85, *p*=0.010).

**Table 4.** Characteristics of those who obtained an antimicrobial without a prescription vs those who did not in northern Tanzania, 2018 (*N*=718)

	Obtained antimicrobial without a prescription, *n*(%) (*N*=85)	Did not obtain antimicrobial without prescription, *n*(%) (*N*=633)	OR (95% CI)	*p*-value^a^
Female	52 (61.2%)	433 (68.4%)	0.73 (0.46, 1.16)	NS
Urban residence	24 (28.2%)	131 (20.7%)	1.51 (0.91, 2.51)	NS
Post-primary education	32 (37.6%)	149 (23.5%)	1.96 (1.22, 3.16)	0.005*
Have health insurance	31 (36.5%)	199 (31.4%)	1.25 (0.78, 2.01)	NS
Christian	72 (84.7%)	512 (80.9%)	1.31 (0.70, 2.44)	NS
Chagga tribe	67 (78.8%)	468 (73.9%)	1.31 (0.76, 2.27)	NS
Prefers pharmacy for febrile syndrome	32 (37.6%)	156 (24.6%)	1.85 (1.15, 2.97)	0.010*
	Obtained antimicrobial without a prescription, mean (sd) (*N*=85)	Did not obtain antimicrobial without prescription, mean (sd) (*N*=633)		*p*-value^b^
Age, years	43.1 (16.4)	48.7 (18.2)		0.007*
SES score	0.42 (0.33)	0.34 (0.29)		0.023*

SES: socioeconomic status; NS: not statistically significant (*p*≥0.05)

*p<0.05

^a^Associations between categorical variables assessed via Pearson’s chi-squared

^b^Associations between categorical and continuous variables assessed via Student’s t-test

## Discussion

This paper describes the prevalence of self-treatment with both antimicrobial and non-antimicrobial medications in an area of low malaria transmission intensity in northern Tanzania. By self-report, self-treatment with antimicrobials was uncommon and the use of antihypertensives and antihyperglycemics without a prescription was rare. The vast majority of respondents who acknowledged obtaining medications without prescriptions reported that they self-selected the medication. A higher level of education, higher SES and younger age were associated with self-treatment, and those who endorsed self-treatment were also more likely to prefer seeking care at pharmacies for febrile illnesses.

The proportion of study participants reporting self-treatment with an antimicrobial in the preceding year was substantially lower than what has been reported elsewhere in sub-Saharan Africa. For example, in a recent study in Sudan that also relied on respondent self-report, 74% of adults reported use of an antimicrobial without a prescription within the preceding month.^20^ Other studies performed in other settings with high malaria prevalence in sub-Saharan Africa have found similarly high prevalences of antimicrobial self-treatment.^1,4,6^ The findings of our study suggest that self-treatment with antimicrobials generally, not just antimalarials, may be substantially less common in areas of low-malaria prevalence. Other possible explanations for lower prevalence of self-treatment in northern Tanzania include sociocultural influences, more effective regulation of dispensation of medications, and more abundant access to healthcare in this setting. Further research is needed to determine the prevalence of self-treatment in other settings with low malaria prevalence and to explore factors contributing to low self-medication rates in northern Tanzania.

Of the antimicrobials, antihelmintics were the most frequently obtained without a prescription in this study. Antihelmintics have not been a focus of previous research regarding self-treatment in sub-Saharan Africa, but the findings presented here suggest the use of antihelmintics without a prescription may be a regional phenomenon worth studying further. Tanzanian pharmaceutical regulations do not require a prescription for most oral antihelmintics. Antihelmintic mass drug administration (MDA) campaigns, which are common in the Kilimanjaro Region and across sub-Saharan Africa, may also have normalized such self-treatment outside of MDA programs. Self-medication with antihypertensives and antihyperglycemics was fortunately extremely rare in this study population, reported by less than 1% of all participants. Further study of the use of these medications without prescriptions is warranted in other settings where self-treatment is more common.

The primary risk factors for self-medication with antimicrobials in this northern Tanzanian population were younger age and increased education. This finding is consistent with other studies from sub-Saharan Africa which also found self-treatment to be associated with greater levels of education and younger age.^6,20^ To maximize effectiveness, educational initiatives to combat inappropriate self-medication with antimicrobials in sub-Saharan Africa should be designed to target the younger, more educated population. Interestingly, self-medication with antimicrobials was associated with higher SES in this study, suggesting that financial concerns about physician and hospital charges may not have been an important driver of self-treatment in this community. Respondents who reported self-treatment with antimicrobials were also significantly more likely to identify a pharmacy as the first place they would seek care for a febrile illness. This link between care-seeking behavior and self-treatment suggests that educational programming regarding the risks of inappropriate self-medication with antimicrobials ought to include information about the appropriate use of pharmacies.

Our study had several limitations. First, like other investigations of self-treatment in sub-Saharan Africa, this study relied on respondent self-report, which is subject to social desirability bias. If participants felt embarrassed to admit obtaining medications without prescriptions, this may have resulted in underreporting of the true rate of self-treatment. Similarly, participants’ responses were subject to recall bias. If respondents forgot certain instances of self-treatment or were unaware of self-treatment episodes by other members of the household, this may also have resulted in an underestimation of the self-medication rate. Furthermore, this study also depended on participants’ ability to correctly distinguish between different kinds of medication. Respondents may, for example, have incorrectly identified an antibacterial as a cold medication or an antihelmintic as an antimalarial. However, our study design was similar to those used in other studies of self-medication in sub-Saharan Africa, which were likely subject to the same biases. Finally, it should be noted that this survey was administered to all eligible households without regard to history of illnesses in the preceding year, potentially resulting in the inclusion of households in which no member had any reason to self-medicate in the preceding year. This approach, however, is consistent with the methods of other community surveys of self-medication in sub-Saharan Africa.^1,3,5^ Moreover, we suspect that the vast majority of households in our study would have had at least one member who had some kind of illness in the preceding 12 months.

In conclusion, self-treatment with both antimicrobials and other prescription medicines such as antihypertensives and antihyperglycemics was uncommon in an area of northern Tanzania with low prevalence of malaria. Further research is needed to explore factors that contribute to low prevalence of self-treatment in this area relative to what has been observed elsewhere in sub-Saharan Africa.

## Supplementary Material

Supplementary Data
