# Supplementary figures and images for: Self-medication with non-prescribed pharmaceutical agents in an area of low malaria transmission in northern Tanzania: a community-based survey
Source: Trans R Soc Trop Med Hyg. 2018 Dec 31;113(4):183–8. doi: 10.1093/trstmh/try138 (PMC6432801; doi:10.1093/trstmh/try138)

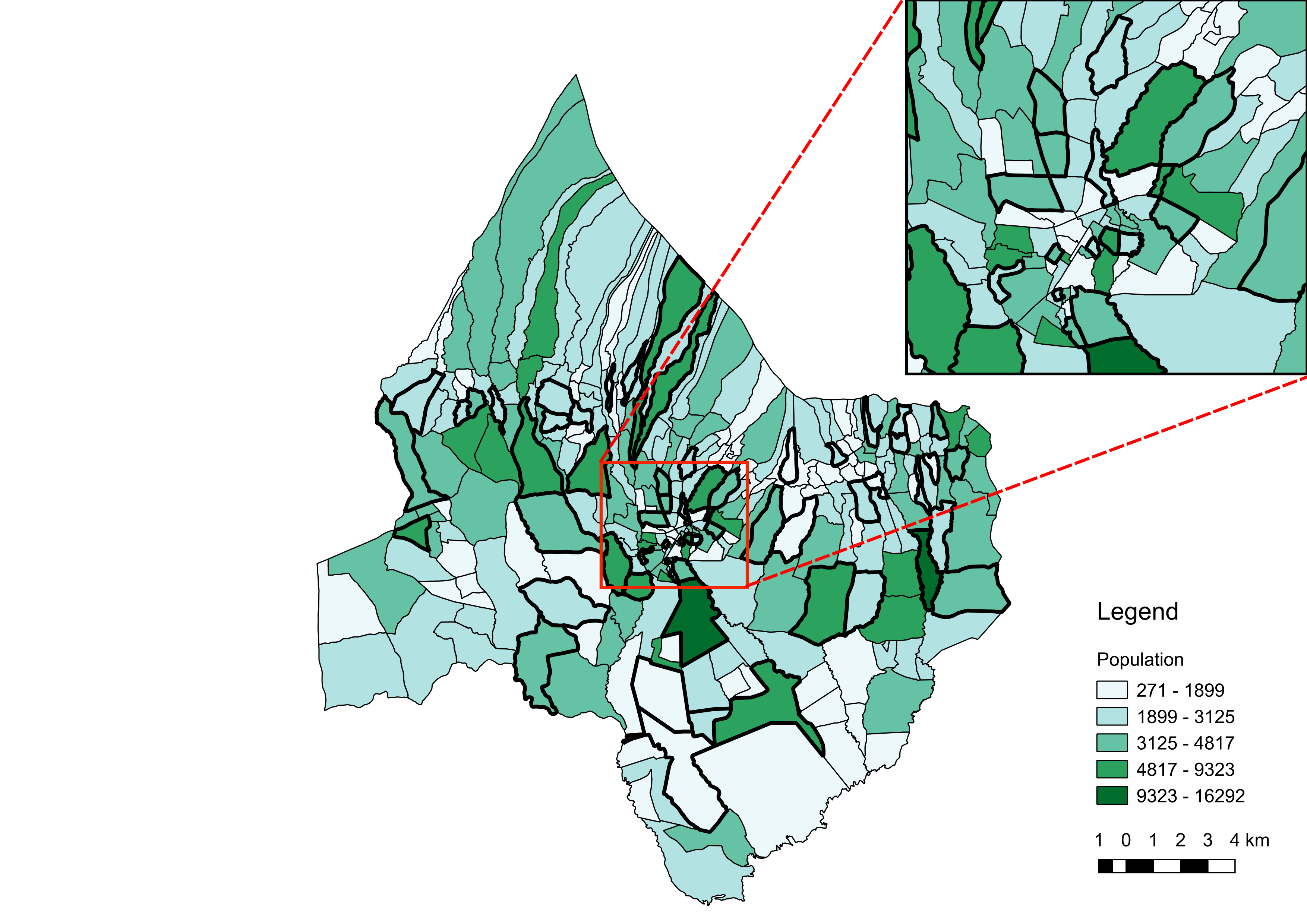

Supplement: Supplementary Data [file try138_supplemental_figure_1.png]
